# MTA1—a stress response protein: a master regulator of gene expression and cancer cell behavior

**DOI:** 10.1007/s10555-014-9525-1

**Published:** 2014-10-21

**Authors:** Rui-An Wang

**Affiliations:** 1 State Key Lab for Cancer Biology, Department of Pathology, Xijing Hospital, Xi’an, China; 2 Department of Pathology and Pathophysiology, The Fourth Military Medical University Xi’an, Xi’an, 710032 China

**Keywords:** MTA1, Stress protein, Carcinogenesis, Metastasis, Hypoxia, Immune stress, Epithelial stem cell misplacement, Apoptosis

## Abstract

Gene mutation’s role in initiating carcinogenesis has been controversial, but it is consensually accepted that both carcinogenesis and cancer metastasis are gene-regulated processes. MTA1, a metastasis-associated protein, has been extensively researched, especially regarding its role in cancer metastasis. In this review, I try to elucidate MTA1’s role in both carcinogenesis and metastasis from a different angle. I propose that MTA1 is a stress response protein that is upregulated in various stress-related situations such as heat shock, hypoxia, and ironic radiation. Cancer cells are mostly living in a stressful environment of hypoxia, lack of nutrition, and immune reaction attacks. To cope with all these stresses, MTA1 expression is upregulated, plays a role of master regulator of gene expression, and helps cancer cells to survive and migrate out of their original dwelling.

## Introduction

Metastasis is the primary cause of cancer-related death. In the past half century, paramount efforts have been made to elucidate the mechanisms involved in cancer metastasis, especially molecular mechanisms, with an aim to design drugs that can block metastasis and thus reduce cancer-caused death. Hundreds of molecules are closely related to metastasis. MTA1 is one that attracts widespread attention for its close relationship with cancer progression, metastasis, and its fantastic role in many other cellular processes. While MTA1 research and review articles are mounting, they still lack insight about what stimulates MTA1 expression and why its overexpression drives metastasis, such as a biological meaning behind all these phenomena. I here present a new carcinogenesis theory viewpoint, stem cell misplacement theory (SCMT) [1], which explains why cancer occurs and metastasizes. We may have a glimpse of MTA1’s role in carcinogenesis and cancer metastasis, not from a mechanistic but a biological point of view.

## Carcinogenesis by stem cell misplacement—carcinoma cells are strayed epithelial cells in the stroma

The traditional view of carcinogenesis as a result of accumulated gene mutation faces increasing challenges [1–5] and evidence falsifying the somatic mutation theory (SMT) is emerging. First, intensive cancer genome studies failed to reveal any specific gene mutation combinations as the cause of cancer. Second, most chemical carcinogens are not genotoxic [6], and those which are genotoxic are not necessarily carcinogenic, such as the famous anti-TB drug isoniazide. Third, increasing evidence shows that most high occurrence gene mutations in cancer cells are associated with better clinical outcomes, which means gene mutations lower cancer malignancy. For example, IDH1 and IDH2 mutations are associated with better glioma patient prognosis [7–9], and Braf mutations are associated with better prognosis in acral lentiginous melanoma [10].

### The possible path from normal epithelial cells to invasive cancer

In humans, around 80–90 % of malignant tumors are epithelially derived carcinomas. Ever since Dr. Broaders first systemically described the *in situ* carcinoma lesion in 1932 [11], the lesion has been seen as the earliest form of cancer. With further morphological observations, the stepwise carcinogenesis model was gradually accepted by the scientific field. This model asserts that an epithelial cell is malignantly transformed due to gene mutation, further proliferates to form atypical hyperplasia, progresses to *in situ* carcinoma, and with gene mutation accumulation, it breaks down the basement membrane separating the epithelium from the connective stroma. It becomes invasive cancer in the stroma, where it can metastasize to distant sites by lymphatics or blood vessels [12]. The model was widely accepted and was taken as fact.

However, this model has never been extensively tested, and its dominance hinders researchers from thinking otherwise, i.e., normal epithelial cells displaced to the connective tissue stroma sites and developed into cancer in the wrong environment. The basic difference between these two models is that the classic model posits that epithelial cells malignantly transform first and then enter the stroma by a process called epithelial-mesenchymal transition (EMT), while the alternative model states that the epithelial cells enter the stroma first and then transform to cancer cells in the wrong environment [1]. With these two possible choices, logically, we cannot prove one model is right unless we prove the other is wrong.

### Paradoxes in the classic model of *in situ* carcinoma to invasive carcinoma

Unfortunately, this exclusive study approach has never been applied to test the classic carcinogenic model. Morphological observations provide support but not evidence for the model *per se*. Since the alternative model has never been studied, we cannot say it is wrong. Interestingly, the classic carcinogenesis model has been studied for many decades, so we should be able to falsify it if it was wrong. In fact, paradoxes falsifying the *in situ* carcinoma to invasive carcinoma model are accumulating and urging us to take a different stance.

The paradoxical evidence comes from different levels. The first evidence level is of molecular pathology [1]. HER2 is a well-known oncogene often amplified and overexpressed in breast cancer. Intriguingly, ductal carcinoma *in situ* (DCIS), which is deemed to be the precursor lesion of invasive ductal cancer, has a much higher rate (50–60 %) of HER2 amplification and overexpression than that of invasive breast cancer, which is about 25 % positive for HER2 [13–15]. Yet, we cannot say that HER2 inhibits DCIS progression to invasive ductal carcinoma. The second level of evidence came from histological pathology. Lobular carcinoma *in situ* (LCIS) and invasive lobular carcinoma (ILC) are both characterized by e-cadherin expression loss, and LCIS is thought to be the precursor lesion of ILC. Paradoxically, if simple LCIS was diagnosed, no specific treatment was needed, since it has been proven that LCIS did not necessarily further progress [16]. Where ILC comes from remains unclear. The third level of evidence includes clinical epidemiology. Evidence has shown that if DCIS was left untreated, only 20 % of patients would develop invasive breast cancer in 10 years [17]. By this speed, if all invasive breast cancer derived from DCIS, its incidence should be many times that of invasive breast cancer, but the opposite is true. Invasive breast cancer incidence is four times that of DCIS [18].

With this evidence, we concluded that not all invasive breast cancer is derived from *in situ* carcinoma [1]. There must be an alternative carcinogenesis path that creates epithelial-derived invasive cancer.

### Carcinogenesis by stem cell misplacement

The above described evidence strongly suggests that the stepwise carcinogenesis model of *in situ* carcinoma to invasive breast cancer is logically impossible [19]. This implies that carcinoma must be grown out *de novo* from the stroma, i.e., developed from the displaced epithelial cells. The SCMT we proposed solved the above puzzle [1]. SCMT posits that carcinoma originates from normal/non-transformed epithelial stem cells displaced in the stroma by the damaged basement membrane (BM) [1]. All known carcinogenic factors, such as inflammation and chronic injury, can damage the BM. Historically, German pathologist Julius Cohnheim suggested carcinogenesis by displaced embryonic stem cells some 150 years ago [20].

**Fig. 1:**
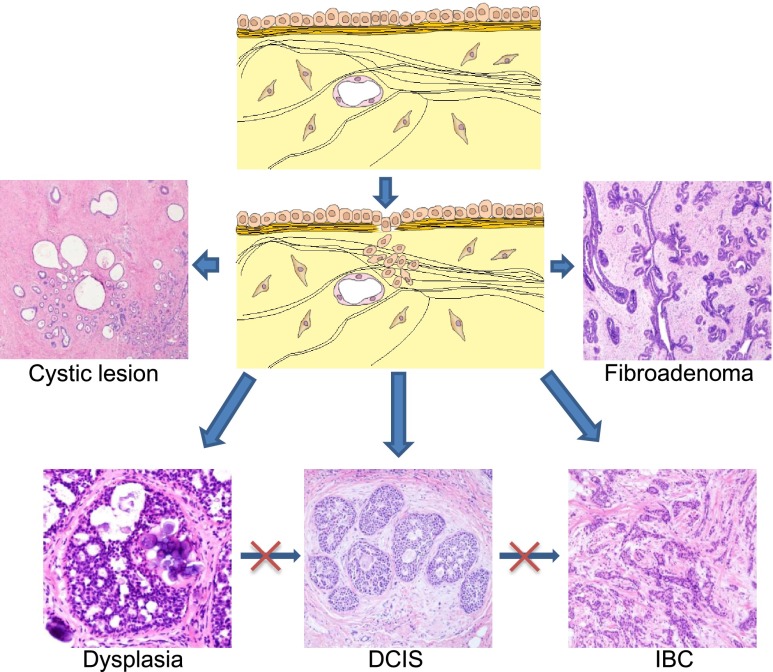
Carcinogenesis by stem cell misplacement. The displaced epithelial cells by the damage of basement membrane have a potential to develop into different benign or malignant lesions as shown in the figure. Dysplasia, in situ carcinoma such as DCIS, and invasive carcinoma are distinct lesion entities, instead of different developmental stages of the same lesion

The current question is not whether the epithelial stem cells can be displaced to the stroma, but regards the fate of the misplaced epithelial cells in the wrong environment. In most cases, we would expect that the misplaced cells die out. However, some could manage to survive. Since they are epithelial cells by nature, they will form epithelial structure types. Usually, if they can differentiate and form BM, they are benign structures like a cyst, a benign tumor, or even normal glandular tissues. However, if they failed to differentiate and form BM, they are carcinoma, i.e., cancer (Fig. 1). The MCF-DCIS cell line is an interesting example that proves the above hypothesis principle. This cell line was derived from the benign MCF10A cell line. When injected into the mammary fat pad of nude mice, it formed DCIS, meaning there is myoepithelial cell differentiation and basement membrane formation [21].

### Survival pressure drives cancer cells to proliferate and metastasize

**Fig. 2:**
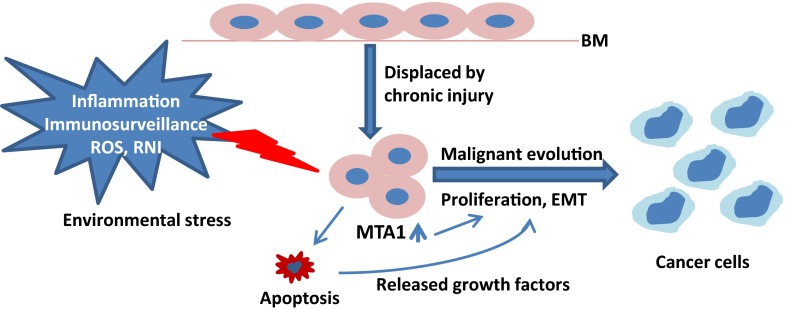
The role of MTA1 in carcinogenesis. By the SCMT model, epithelial cells are displaced to the stromal tissue. The environmental stresses such as inflammation, immunosurveillance, reactive oxygen species (ROS), reactive nitrogen intermediates (RNI), stimulate the expression of MTA1, which in turn promotes the malignant transformation, proliferation, and EMT of the misplaced epithelial cells. Pay attention that apoptosis also plays a positive role in the process of carcinogenesis instead of being a barrier

The primary cause for epithelial cells to transform to cancer cells in the stroma is survival pressure (Fig. 2). The misplaced epithelial cells in the stroma are in a stressful state. Compared to the epithelium microenvironment, the stroma is a place of hyperoxia and rich in immune cells, antibodies, and other immune-related cytokines. Moreover, the situations introducing epithelial cell displacement to the stroma are often associated with inflammatory reactions. The misplaced epithelial cells must fight for their existence. Interestingly, increasing population is an effective way of maintaining existence. Therefore, the higher the environmental pressure and greater the cell death, the faster the misplaced epithelial cells must grow [19, 22]. Also, pathologists saw that increased apoptosis was associated with higher cancer malignancy and poor clinical outcome [22–28].

Although it is still widely believed that resistance to apoptosis is a hallmark of cancer [29, 30], the evidence favors the opposite view [19, 22, 23]. So far, there are no documented carcinogenic agents that can promote cell survival. Instead, they are mostly cytotoxic and induce cell death. For example, aflatoxin and various viruses whose infection induces liver cancer all induce liver cell death. The HBV virus X protein is the most potent factor of the HBV virus’s carcinogenic effects and is an apoptosis-inducing protein [31–38]. Of the other known apoptosis-inducing genes, such as the cell death receptor CD95 and death executor protein Caspase-3, all are known to promote tumor growth [39, 40]. Conversely, anti-apoptosis factors inhibit carcinogenesis and cancer growth. Autophagy inhibits apoptosis and carcinogenesis [41]. Bcl-2, the anti-apoptotic protein prototype, inhibits carcinogenesis and cancer cell growth both *in vitro* and *in vivo* [42–44]. In various cancers, including breast cancer, colon cancer, and non-small cell lung cancer, Bcl-2 overexpression is a predicting factor of favorable clinical outcomes [45–51].

Inducing apoptosis as a therapeutic strategy has been touted for the past two decades in both academics and industrial labs without much success. A recent study showed that the IAP inhibitor, which was developed to treat cancer, promotes breast cancer metastasis to bone [52]. Similarly, anti-angiogenic agents and radiotherapy were all found to stimulate cancer metastasis [53–55]. Therefore, metastasis is a basic response of cancer cells to stress [56].

### EMT as a camouflage

Epithelial mesenchymal transition has been extensively studied in cancer metastasis research over the past decade. Most studies focused on the mechanisms and signaling pathways involved in EMT with the aim of targeting therapy. However, people rarely asked why cancer cells would ever start EMT. Obviously, the notion of *in situ* carcinoma progressing to invasive cancer by EMT does not hold up, as the invasive cancer does not derive from the *in situ* carcinoma. LCIS is characterized by e-cadherin expression loss [16], an EMT hallmark. Ironically, it has been clinically proven that LCIS lesions do not further develop and do not need special treatment [16].

I propose that EMT is a way of immune escape. We know that by nature, carcinoma consists of epithelial cells trapped in mesenchymal tissue. The mesenchymal tissue is not the home of epithelial cells. These epithelial cancer cells thus become the easy target of the immune system. Interestingly, although immune response against cancer has been found for almost six decades [57], there have been no cancer-specific antigens identified for most cancer types. Though the issue has not been explored before by immunologists, I believe that epithelial cell invasion to mesenchyme would provoke an immune response, and the antigen might be the epithelial marker that discriminates cancer cells from the surrounding mesenchymal cells. Thus, to lower the risk of being targeted, the cancer cells would reduce epithelial marker expressions, and, as an adaptation response, express some mesenchymal cell-type proteins. This is in accordance with the biosphere law. We see that jungle animals exhibit colors and patterns similar to their environment to lower their chances of being targeted. Therefore, EMT is a way of immune escape by the strategy of camouflage (Fig. 2).

### Molecular adaptations during carcinogenesis progression

Adaptation is an important pathology concept and a general biosphere phenomenon. The esophageal epithelium is stratified squamous epithelium, which is resistance to wear and tear but not resistant to acid. Therefore, when gastric acid reflux happens often, the epithelium of the lower part of the esophagus would turn to columnar epithelium, which is more resistant to gastric acid. This is termed metaplasia, a form of pathological adaptation. Similarly, we proposed the concept of molecular adaptation [58]. The molecular adaptations include adaptive mutations and adaptive epigenetic modifications. The former includes point mutations, amplifications, and deletions.

The concept of adaptive mutation was proposed by Cairns three decades ago [59] and has been a controversial issue since then. Most contemporary molecular geneticists are New Darwinists and hold that gene mutations are stochastic in nature. They do not believe in adaptive mutation. It is true that we do not know the adaptive mutation mechanism, but that does not mean it does not exist. We can use the Braf V600E mutation in nevus cells as an example. Around 80 % of nevus cells have this point mutation [60]. Obviously, we cannot explain this phenomenon as a random mutation. Cell cycle regulators are also good examples of molecular adaptions. It is well known that cyclins are often overexpressed and cyclin-dependent kinases (CDKs) are over-activated in cancer, yet the cancer cell proliferation cycle duration is not shorter than the corresponding normal cells but prolonged or showing no change [61]. This paradox is explained by molecular adaptation. The prolonged cell cycle means there is increased resistance and thus requires more cyclins and more active CDKs. Otherwise, cells cannot divide.

## MTA1 is a stress response protein

**Fig. 3:**
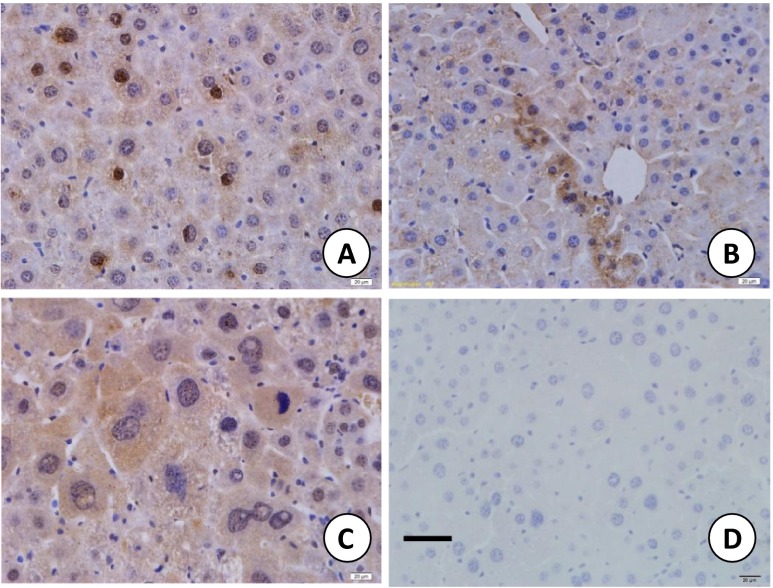
MTA1 expression in a dimethylnitrosamine-induced mouse liver carcinogenesis model. Dimethynitrosamine was given at a dose of 100 mg/Kg body weight by gavage, and 0.1 ml of 20 % of CCl_4_ in olive oil was given after 3 days by gavage, twice per week. MTA1 expression was upregulated, and more obviously seen in the cytoplasm. This suggests that MTA1 also functions in the cytoplasm in stress. *A* control, *B* 60 days, *C* 150 days after treatment. *D* negative staining control of a tissue slide from a mouse of 150 days after treatment. *Bar* = 30 μm

MTA1 was initially isolated from highly invasive breast cancer cell lines, and its expression was associated with cancer progression and metastasis in a variety of human cancers [62–64] However, the factor responsible for upregulating MTA1 in cancer was unknown until Mazumdar *et al.* found that heregulin, a ligand for HER3, was capable of inducing MTA1 expression [65]. It was later discovered that hypoxia, ironic radiation, inflammation, as well as heat shock all strongly upregulated MTA1 expression [65–73]. Since hypoxia, ironic radiation, and heat shock are all stress agents, we may conclude that MTA1 is a stress response protein. Its expression in the adverse and fluctuating immediate cancer cell surroundings may help survival in harsh conditions and escape from danger. In many stress conditions, such as trauma and inflammation, growth factors are released. Therefore, heregulin-stimulated MTA1 expression also falls in this stress response category (Figs. 2 and 3).

### Stress response proteins are upregulated in cancer

As described above, carcinoma cells live in a stressful environment quite different from the epithelium. Initially, when epithelial cells just land to the stroma, it is hyperoxic, with ample reactive oxygen species and reactive nitrogen intermediates, immune cells, and cytokines. When they proliferate and grow, it is hypoxic due to lack of blood supply. Therefore, most, if not all, stress response proteins are highly expressed in cancer cells. For example, heat shock proteins, hypoxia inducible factors, and MAPK kinases such as p38MAPK, MAPK13, p53, and MTA1 are all stress-related proteins and proposed therapeutic cancer targets.

### Induction of MTA1 expression by heat shock, hypoxia, irradiation, and X protein of hepatitis B virus and dimethylnitrosamine

The first factor known to be able to stimulate MTA1 expression in breast cancer cells is the growth factor heregulin [74]. We know that growth factors are often released during trauma, so heregulin can be regarded as a stress-related factor. Korean scientists later found that hypoxia induced MTA1 expression, which helped stabilize HIF1α by recruiting histone deacetylase complex 1(HDAC1) [69]. Since HIF1a plays an important role in angiogenesis and promotion of cancer metastasis, MTA1 also has a part to play in both the normal wound healing and cancer. Li *et al.* further found that ionic radiation induced marked elevation of MTA1 protein expression in U2OS osteosarcoma cells, mammary glands, thymus, and skin of mice [70]. The increased amount of MTA1 helps stabilize p53 and thus plays a role in repairing damaged DNA [71]. Moreover, MTA1 protein levels were elevated in a germ cell tumor cell line after heat shock and protected the cell from heat shock-induced apoptosis [68]. The X protein of hepatitis B virus is generally believed to be responsible for the virus’s carcinogenic effect [75]. It promotes both liver cell apoptosis and proliferation. Interestingly, X protein strongly induced MTA1 protein expression [66, 76]. Dimethynitrosamine (DEN) has a strong toxicity to the liver. Long-term treatment of rodents with DEN can induce liver cancer. We found that along with the increased liver cell damage, the MTA1 expression level was also increased not only in the nuclei but in the cytoplasm (Fig. 3) [77].

## MTA1 overexpression is associated with unfavorable prognosis

There are numerous paradoxes in our current knowledge about cancer. Although it is widely believed that cancer is the result of accumulated gene mutations, many of these mutations are associated with better clinical outcomes. For example, IDH1 and IDH2 mutations are associated with much better glioma patient prognosis, and the BRAF mutation was associated with more favorable acral lentiginous melanoma prognosis [10]. More interestingly, although apoptosis is taken as a barrier to carcinogenesis and resistance to apoptosis is regarded as a hallmark of cancer, the overexpression of the antiapoptotic oncogene Bcl-2 was an indicator of favorable prognosis in breast cancer, colon cancer, and non-small cell lung cancers. However, MTA1 overexpression was unanimously associated with more advanced cancer stages, increased metastasis tendency, and unfavorable outcomes [78, 79]. So far, the reported correlation between MTA1 overexpression and cancer progression and prognosis includes breast cancer [80–83], colon cancer [63, 84], esophageal cancer [64, 85], lung cancer [86, 87], liver cancer [88, 89], gastric cancer [63], thymoma [90], ovarian cancer [91, 92], nasopharyngeal cancer [93, 94], pancreatic cancer [95, 96], prostate cancer [97, 98], and chorionic carcinoma [99].

## Biological functions of MTA1

Though MTA1 is described as a stress response protein, how it helps cells in environmental stress remains unclear. Though its functional roles are still elusive, many targeting genes and collaboration partners have been identified at MTA1 downstream.

### Functions at the molecular level—regulation of gene expression by both affecting protein transcription and stabilization

After MTA proteins were found to be a component of nucleosome remodeling and the deacetylation (NuRD) complex, many downstream targets were discovered. Mazumdar *et al.* first found that MTA1 inhibits ER transactivation activity by recruiting HDAC2 to the promoters of ER targeting genes [74]. It was later found that MTA1 binds transcription factor Six3 [100] and in a negative feedback fashion inhibits Six3 expression and its downstream targets [101]. Paradoxically, MTA1 was also found to be a coactivator protein [102]. By binding and recruiting Pol II and C-Jun to the FosB promoter, MTA1 stimulates FosB expression [102]. Further, MTA1 binds FosB in the E-cadherin promoter region and recruits HDAC2 to downregulate E-cadherin expression, a hallmark of the epithelial mesenchymal transition [102]. Since ER, Six3, FosB, and possibly many other transcription factors regulate the expression of a broad spectrum of genes, MTA1 may thus exert a wide range of regulatory functions.

Except for functioning as a transcriptional coregulator, MTA1 was also found to stabilize proteins by directly binding to them and inhibiting their break down through ubiquitination inhibition. For example, the expressions of both MTA1 and p53 were upregulated in cells exposed to ionizing radiation [71]. MTA1 was found to bind and stabilize p53, which is notoriously known to have a very short half-life [71]. Similarly, in cells cultured under hypoxic conditions, MTA1 and HIF1α expression were both upregulated, and MTA1 bonded to and stabilized HIF1α. They collaborate to promote angiogenesis and may improve the condition of nourishment and oxygen supply [68].

### Functions at the cellular level

MTA1 was found to block p53-induced apoptosis [103], induce cell proliferation [101], and promote epithelial mesenchymal transition in different studies [102]. However, promotion of cell survival is not positively linked with cell proliferation. In fact, in most cases, or by principle, apoptosis reduction and cell proliferation are negatively correlated [19]. The less apoptosis, the slower the cell grows [19]. Bcl-2, the typical anti-apoptotic protein, inhibits cell growth [43] as well as p202, an interferon-induced antiapoptotic protein [104, 105]. Conversely, CD95, caspase 3, and HBVx, all induce apoptosis and promote tumor cell growth [39, 40, 75]. The case of MTA1 is more complex. Environmental stress stimulates its expression, which may protect cells from apoptosis in a certain context, but is still not enough to make cells live long in an adverse environment. Therefore, cells may still show increased proliferation. As for EMT and metastasis, it is quite natural that MTA1 mediates these processes, but it cannot be the only molecule. Without MTA1, cells would still be able to migrate and invade, though perhaps be compromised.

### Functions at the organismal level

Studies from MTA1 gene-modified mice have revealed a large range of functions MTA1 may play. MTA1 was found to play roles in circadian rhythm maintenance [106], embryonic development regulation, and visual performance by regulating rhodopsin expression [102]. A reduced rate of breast cancer metastasis to lung was observed in the MTA1 null genetic background [107]. More functions of MTA1 at body level are expected to be revealed by gene-modified animal studies.

## Conclusion

MTA1 is a key factor in cancer metastasis, and its overexpression was consistently found to be associated with cancer’s advanced stages, higher malignancy degree, and poorer patient prognosis. The biological meaning behind these phenomena remains unknown. By using stem cell misplacement theory, I interpreted that carcinoma are developed from misplaced epithelial stem cells in the stressful wrong stroma environment, which is often affected by subtle, chronic inflammation. I proposed that MTA1 is a stress response protein, like heat shock protein, hypoxia-inducing factors, and p53. MTA1 overexpression helps cells cope with environmental stressors like hypoxia or hyperoxia, hyperthermia, immune reactions, and possibly radiation and chemotherapies, which would increase their chances of survival in the adverse environment. MTA1 overexpression stabilizes both HIF1α and p53, which both play important roles in carcinogenesis.

Both carcinogenesis and cancer metastasis are rather complex issues, though, and so is MTA1’s role in these processes. Metastasis is a cancer cell stress response, and MTA1 as a stress protein is a stress level indicator. Therefore, it is no surprise that MTA1 overexpression correlates well with cancer metastasis and is often an indicator of poor prognosis, no matter it has a role in metastasis or not. Conversely, as many studies have shown, MTA1 does play a role in helping cancer cells coping with stress by increasing their survival, angiogenesis, migration and invasion abilities, and epithelial mesenchymal transition in collaboration with other stress proteins such as HIF1α, p53, and TGFR. Though it appears to be an attractive target for blocking cancer metastasis, it may not be that promising. Of the molecules involved in cancer metastasis, MTA1 is an important one but certainly not the only. The force driving cancer metastasis is stress, and the struggle for existence and MTA1 overexpression is a sign of these stresses.

## References

[1] Wang RA, Li ZS, Zhang HZ, Zheng PJ, Li QL, Shi JG (2013). Invasive cancers are not necessarily from preformed in situ tumours—an alternative way of carcinogenesis from misplaced stem cells. Journal of Cellular and Molecular Medicine.

[2] Baker, S. G. (2012/2013). Paradox-driven cancer research. *Disruptive Science and Technology, 1*, 143–148.

[3] Soto AM, Sonnenschein C (2011). The tissue organization field theory of cancer: a testable replacement for the somatic mutation theory. Bioessays.

[4] Duesberg P (2005). Does aneuploidy or mutation start cancer?. Science.

[5] Meng X, Zhong J, Liu S, Murray M, Gonzalez-Angulo AM (2012). A new hypothesis for the cancer mechanism. Cancer Metastasis Review.

[6] Weinberg RA (2014). Coming full circle-from endless complexity to simplicity and back again. Cell.

[7] Turkalp Z, Karamchandani J, Das S (2014). IDH Mutation in.

[8] Gorovets D, Kannan K, Shen R, Kastenhuber ER, Islamdoust N, Campos C (2012). IDH mutation and neuroglial developmental features define clinically distinct subclasses of lower grade diffuse astrocytic glioma. Clinical Cancer Research.

[9] Qi ST, Yu L, Lu YT, Ou YH, Li ZY, Wu LX (2011). IDH mutations occur frequently in Chinese glioma patients and predict longer survival but not response to concomitant chemoradiotherapy in anaplastic gliomas. Oncology Reports.

[10] Hong JW, Lee S, Kim DC, Kim KH, Song KH (2014). Prognostic and clinicopathologic associations of BRAF mutation in primary acral lentiginous melanoma in Korean patients: a preliminary study. Annals of Dermatology.

[11] Broders AC (1932). Carcinoma in situ contrasted with benign penetrating epithelium. JAMA-Journal of The American Medical Association.

[12] Burstein HJ, Polyak K, Wong JS, Lester SC, Kaelin CM (2004). Ductal carcinoma in situ of the breast. New England Journal of Medicine.

[13] Latta EK, Tjan S, Parkes RK, O’Malley FP (2002). The role of HER2/neu overexpression/amplification in the progression of ductal carcinoma in situ to invasive carcinoma of the breast. Modern Pathology.

[14] Barnes DM, Bartkova J, Camplejohn RS, Gullick WJ, Smith PJ, Millis RR (1992). Overexpression of the c-erbB-2 oncoprotein: why does this occur more frequently in ductal carcinoma in situ than in invasive mammary carcinoma and is this of prognostic significance?. European Journal of Cancer.

[15] Allred DC, Clark GM, Molina R, Tandon AK, Schnitt SJ, Gilchrist KW (1992). Overexpression of HER-2/neu and its relationship with other prognostic factors change during the progression of in situ to invasive breast cancer. Human Pathology.

[16] Frykberg ER (1999). Lobular carcinoma in situ of the breast. Breast Journal.

[17] Sanders ME, Schuyler PA, Dupont WD, Page DL (2005). The natural history of low-grade ductal carcinoma in situ of the breast in women treated by biopsy only revealed over 30 years of long-term follow-up. Cancer.

[18] Virnig BA, Wang SY, Shamilyan T, Kane RL, Tuttle TM (2010). Ductal carcinoma in situ: risk factors and impact of screening. Journal of National Cancer Institute Monographs.

[19] Wang RA, Li ZS, Yan QG, Bian XW, Ding YQ, Du X (2014). Resistance to apoptosis should not be taken as a hallmark of cancer. Chinese Journal of Cancer.

[20] Baker SG (2012). Paradoxes in carcinogenesis should spur new avenues of research: an historical perspective. Disruptive Sciences and Technology..

[21] Miller FR, Santner SJ, Tait L, Dawson PJ (2000). MCF10DCIS.com xenograft model of human comedo ductal carcinoma in situ. Journal of National Cancer Institute.

[22] Wang RA, Li QL, Li ZS, Zheng PJ, Zhang HZ, Huang XF (2013). Apoptosis drives cancer cells proliferate and metastasize. Journal of Cellular and Molecular Medecine.

[23] Lipponen P, Aaltomaa S, Kosma VM, Syrjanen K (1994). Apoptosis in breast cancer as related to histopathological characteristics and prognosis. European Journal of Cancer.

[24] Lipponen PK, Aaltomaa S (1994). Apoptosis in bladder cancer as related to standard prognostic factors and prognosis. Journal of Pathology.

[25] Zhang GJ, Kimijima I, Abe R, Watanabe T, Kanno M, Hara K (1998). Apoptotic index correlates to bcl-2 and p53 protein expression, histological grade and prognosis in invasive breast cancers. Anticancer Research.

[26] Sinicrope FA, Hart J, Hsu HA, Lemoine M, Michelassi F, Stephens LC (1999). Apoptotic and mitotic indices predict survival rates in lymph node-negative colon carcinomas. Clinical Cancer Research.

[27] Lipponen P (1999). Apoptosis in breast cancer: relationship with other pathological parameters. Endocrine Related Cancer.

[28] Nishimura R, Nagao K, Miyayama H, Matsuda M, Baba K, Matsuoka Y (1999). Apoptosis in breast cancer and its relationship to clinicopathological characteristics and prognosis. Journal of Surgical Oncology.

[29] Hanahan D, Weinberg RA (2000). The hallmarks of cancer. Cell.

[30] Hanahan D, Weinberg RA (2011). Hallmarks of cancer: the next generation. Cell.

[31] Kuo CY, Tsai JI, Chou TY, Hung MJ, Wu CC, Hsu SL (2012). Apoptosis induced by hepatitis B virus X protein in a CCL13-HBx stable cell line. Oncology Reports.

[32] Tang RX, Kong FY, Fan BF, Liu XM, You HJ, Zhang P (2012). HBx activates FasL and mediates HepG2 cell apoptosis through MLK3-MKK7-JNKs signal module. World Journal of Gastroenterology.

[33] Hu L, Chen L, Yang G, Li L, Sun H, Chang Y (2011). HBx sensitizes cells to oxidative stress-induced apoptosis by accelerating the loss of Mcl-1 protein via caspase-3 cascade. Molecular Cancer.

[34] Kim JY, Song EH, Lee HJ, Oh YK, Choi KH, Yu DY (2010). HBx-induced hepatic steatosis and apoptosis are regulated by TNFR1- and NF-kappaB-dependent pathways. Journal of Molecular Biology.

[35] Cheng P, Li Y, Yang L, Wen Y, Shi W, Mao Y (2009). Hepatitis B virus X protein (HBx) induces G2/M arrest and apoptosis through sustained activation of cyclin B1-CDK1 kinase. Oncology Reports.

[36] Niu D, Zhang J, Ren Y, Feng H, Chen WN (2009). HBx genotype D represses GSTP1 expression and increases the oxidative level and apoptosis in HepG2 cells. Molecular Oncology.

[37] Tanaka Y, Kanai F, Kawakami T, Tateishi K, Ijichi H, Kawabe T (2004). Interaction of the hepatitis B virus X protein (HBx) with heat shock protein 60 enhances HBx-mediated apoptosis. Biochemical and Biophysical Research Communication.

[38] Su F, Theodosis CN, Schneider RJ (2001). Role of NF-kappaB and myc proteins in apoptosis induced by hepatitis B virus HBx protein. Journal of Virology.

[39] Chen L, Park SM, Tumanov AV, Hau A, Sawada K, Feig C (2010). CD95 promotes tumour growth. Nature.

[40] Huang Q, Li F, Liu X, Li W, Shi W, Liu FF (2011). Caspase 3-mediated stimulation of tumor cell repopulation during cancer radiotherapy. Nature Medicine.

[41] Sun K, Guo XL, Zhao QD, Jing YY, Kou XR, Xie XQ (2013). Paradoxical role of autophagy in the dysplastic and tumor-forming stages of hepatocarcinoma development in rats. Cell Death and Disease.

[42] Murphy KL, Kittrell FS, Gay JP, Jager R, Medina D, Rosen JM (1999). Bcl-2 expression delays mammary tumor development in dimethylbenz(a)anthracene-treated transgenic mice. Oncogene.

[43] Knowlton K, Mancini M, Creason S, Morales C, Hockenbery D, Anderson BO (1998). Bcl-2 slows in vitro breast cancer growth despite its antiapoptotic effect. Journal of Surgical Research.

[44] de La Coste A, Mignon A, Fabre M, Gilbert E, Porteu A, Van Dyke T (1999). Paradoxical inhibition of c-myc-induced carcinogenesis by Bcl-2 in transgenic mice. Cancer Research.

[45] Yang Q, Sakurai T, Yoshimura G, Suzuma T, Umemura T, Nakamura M (2003). Prognostic value of Bcl-2 in invasive breast cancer receiving chemotherapy and endocrine therapy. Oncology Reports.

[46] Callagy GM, Pharoah PD, Pinder SE, Hsu FD, Nielsen TO, Ragaz J (2006). Bcl-2 is a prognostic marker in breast cancer independently of the Nottingham Prognostic Index. Clinical Cancer Research.

[47] Lee KH, Im SA, Oh DY, Lee SH, Chie EK, Han W (2007). Prognostic significance of bcl-2 expression in stage III breast cancer patients who had received doxorubicin and cyclophosphamide followed by paclitaxel as adjuvant chemotherapy. BMC Cancer.

[48] Rolland P, Spendlove I, Madjd Z, Rakha EA, Patel P, Ellis IO (2007). The p53 positive Bcl-2 negative phenotype is an independent marker of prognosis in breast cancer. International Journal of Cancer.

[49] Poincloux L, Durando X, Seitz JF, Thivat E, Bardou VJ, Giovannini MH (2009). Loss of Bcl-2 expression in colon cancer: a prognostic factor for recurrence in stage II colon cancer. Surgical Oncology-Oxford.

[50] Watson NF, Madjd Z, Scrimegour D, Spendlove I, Ellis IO, Scholefield JH (2005). Evidence that the p53 negative / Bcl-2 positive phenotype is an independent indicator of good prognosis in colorectal cancer: a tissue microarray study of 460 patients. World Journal of Surgical Oncology.

[51] Tomita M, Matsuzaki Y, Edagawa M, Shimizu T, Hara M, Onitsuka T (2003). Prognostic significance of bcl-2 expression in resected pN2 non-small cell lung cancer. European Journal of Surgical Oncology.

[52] Yang C, Davis JL, Zeng R, Vora P, Su X, Collins LI (2013). Antagonism of inhibitor of apoptosis proteins increases bone metastasis via unexpected osteoclast activation. Cancer Discovery.

[53] Ebos JM, Lee CR, Cruz-Munoz W, Bjarnason GA, Christensen JG, Kerbel RS (2009). Accelerated metastasis after short-term treatment with a potent inhibitor of tumor angiogenesis. Cancer Cell.

[54] Paez-Ribes M, Allen E, Hudock J, Takeda T, Okuyama H, Vinals F (2009). Antiangiogenic therapy elicits malignant progression of tumors to increased local invasion and distant metastasis. Cancer Cell.

[55] Bouchard G, Bouvette G, Therriault H, Bujold R, Saucier C, Paquette B (2013). Pre-irradiation of mouse mammary gland stimulates cancer cell migration and development of lung metastases. British Journal of Cancer.

[56] Pani G, Galeotti T, Chiarugi P (2010). Metastasis: cancer cell’s escape from oxidative stress. Cancer Metastasis Review.

[57] PREHN RT, MAIN JM (1957). Immunity to methylcholanthrene-induced sarcomas. Journal National Cancer Institute.

[58] Wang RA, Yan QG (2013). Adaptation biology and medicine.

[59] Cairns J (1980). Efficiency of the adaptive response of Escherichia coli to alkylating agents. Nature.

[60] Wu J, Rosenbaum E, Begum S, Westra WH (2007). Distribution of BRAF T1799A(V600E) mutations across various types of benign nevi: implications for melanocytic tumorigenesis. American Journal of Dermatopathology.

[61] Baserga R (1965). The relationship of the cell cycle to tumor growth and control of cell division: a review. Cancer Research.

[62] Toh Y, Pencil SD, Nicolson GL (1994). A novel candidate metastasis-associated gene, mta1, differentially expressed in highly metastatic mammary adenocarcinoma cell lines. cDNA cloning, expression, and protein analyses. Journal of Biological Chemistry.

[63] Toh Y, Oki E, Oda S, Tokunaga E, Ohno S, Maehara Y (1997). Overexpression of the MTA1 gene in gastrointestinal carcinomas: correlation with invasion and metastasis. International Journal of Cancer.

[64] Toh Y, Kuwano H, Mori M, Nicolson GL, Sugimachi K (1999). Overexpression of metastasis-associated MTA1 mRNA in invasive oesophageal carcinomas. British Journal of Cancer.

[65] Liang Y, Dong Y, Zhao J, Li W (2013). YES1 activation elicited by heat stress is anti-apoptotic in mouse pachytene spermatocytes. Biology of Reproduction.

[66] Bui-Nguyen TM, Pakala SB, Sirigiri RD, Xia W, Hung MC, Sarin SK (2010). NF-kappaB signaling mediates the induction of MTA1 by hepatitis B virus transactivator protein HBx. Oncogene.

[67] Li W, Wu ZQ, Zhao J, Guo SJ, Li Z, Feng X (2011). Transient protection from heat-stress induced apoptotic stimulation by metastasis-associated protein 1 in pachytene spermatocytes. PLoS One.

[68] Li W, Bao W, Ma J, Liu X, Xu R, Wang RA (2008). Metastasis tumor antigen 1 is involved in the resistance to heat stress-induced testicular apoptosis. FEBS Letters.

[69] Yoo YG, Kong G, Lee MO (2006). Metastasis-associated protein 1 enhances stability of hypoxia-inducible factor-1alpha protein by recruiting histone deacetylase 1. EMBO Journal.

[70] Ohshiro K, Reddy SD, Pakala SB, Lee MH, Zhang Y (2009). E3 ubiquitin ligase COP1 regulates the stability and functions of MTA1. Proceedings of the National Academy of Sciences of USA.

[71] Li DQ, Divijendra NRS, Pakala SB, Wu X, Zhang Y, Rayala SK (2009). MTA1 coregulator regulates p53 stability and function. Journal of Biological Chemistry.

[72] Li DQ, Ohshiro K, Khan MN, Kumar R (2010). Requirement of MTA1 in ATR-mediated DNA damage checkpoint function. Journal of Biological Chemistry.

[73] Pakala SB, Bui-Nguyen TM, Reddy SD, Li DQ, Peng S, Rayala SK (2010). Regulation of NF-kappaB circuitry by a component of the nucleosome remodeling and deacetylase complex controls inflammatory response homeostasis. Journal of Biological Chemistry.

[74] Mazumdar A, Wang RA, Mishra SK, Adam L, Bagheri-Yarmand R, Mandal M (2001). Transcriptional repression of oestrogen receptor by metastasis-associated protein 1 corepressor. Nature Cell Biology.

[75] Motavaf M, Safari S, Saffari JM, Alavian SM (2013). Hepatitis B virus-induced hepatocellular carcinoma: the role of the virus x protein. Acta Virologica.

[76] Yoo YG, Na TY, Seo HW, Seong JK, Park CK, Shin YK (2008). Hepatitis B virus X protein induces the expression of MTA1 and HDAC1, which enhances hypoxia signaling in hepatocellular carcinoma cells. Oncogene.

[77] Xin B, Wang XY, Li Y, Qin JH, Ma XJ, Yin JP (2012). Expression and potential role of metastasis-associated protein 1 in the induced carcinogenesis of mouse liver. Xi Bao Yu Fen Zi Mian Yi Xue Za Zhi(Chinese).

[78] Hofer MD, Tapia C, Browne TJ, Mirlacher M, Sauter G, Rubin MA (2006). Comprehensive analysis of the expression of the metastasis-associated gene 1 in human neoplastic tissue. Archives of Pathology and Laboratory Medicine.

[79] Luo H, Li H, Yao N, Hu L, He T (2014). Metastasis-associated protein 1 as a new prognostic marker for solid tumors: a meta-analysis of cohort studies. Tumour Biology.

[80] Jang KS, Paik SS, Chung H, Oh YH, Kong G (2006). MTA1 overexpression correlates significantly with tumor grade and angiogenesis in human breast cancers. Cancer Science.

[81] Zhang H, Stephens LC, Kumar R (2006). Metastasis tumor antigen family proteins during breast cancer progression and metastasis in a reliable mouse model for human breast cancer. Clinical Cancer Research.

[82] Martin MD, Hilsenbeck SG, Mohsin SK, Hopp TA, Clark GM, Osborne CK (2006). Breast tumors that overexpress nuclear metastasis-associated 1 (MTA1) protein have high recurrence risks but enhanced responses to systemic therapies. Breast Cancer Research and Treatment.

[83] Cheng CW, Liu YF, Yu JC, Wang HW, Ding SL, Hsiung CN (2012). Prognostic significance of cyclin D1, beta-catenin, and MTA1 in patients with invasive ductal carcinoma of the breast. Annals of Surgical Oncology.

[84] Higashijima J, Kurita N, Miyatani T, Yoshikawa K, Morimoto S, Nishioka M (2011). Expression of histone deacetylase 1 and metastasis-associated protein 1 as prognostic factors in colon cancer. Oncology Reports.

[85] Li SH, Wang Z, Liu XY (2009). Metastasis-associated protein 1 (MTA1) overexpression is closely associated with shorter disease-free interval after complete resection of histologically node-negative esophageal cancer. World Journal of Surgery.

[86] Sasaki H, Moriyama S, Nakashima Y, Kobayashi Y, Yukiue H, Kaji M (2002). Expression of the MTA1 mRNA in advanced lung cancer. Lung Cancer.

[87] Xu L, Mao XY, Fan CF, Zheng HC (2011). MTA1 expression correlates significantly with cigarette smoke in non-small cell lung cancer. Virchows Archiv: an International Journal of Pathology.

[88] Hamatsu T, Rikimaru T, Yamashita Y, Aishima S, Tanaka S, Shirabe K (2003). The role of MTA1 gene expression in human hepatocellular carcinoma. Oncology Reports.

[89] Moon WS, Chang K, Tarnawski AS (2004). Overexpression of metastatic tumor antigen 1 in hepatocellular carcinoma: relationship to vascular invasion and estrogen receptor-alpha. Human Pathology.

[90] Sasaki H, Yukiue H, Kobayashi Y, Nakashima Y, Kaji M, Fukai I (2001). Expression of the MTA1 mRNA in thymoma patients. Cancer Letters.

[91] Murakami M, Kaul R, Robertson ES (2008). MTA1 expression is linked to ovarian cancer. Cancer Biology & Therapy.

[92] Prisco MG, Zannoni GF, De Stefano I, Vellone VG, Tortorella L, Fagotti A (2012). Prognostic role of metastasis tumor antigen 1 in patients with ovarian cancer: a clinical study. Human Pathology.

[93] Deng YF, Zhou DN, Ye CS, Zeng L, Yin P (2012). Aberrant expression levels of MTA1 and RECK in nasopharyngeal carcinoma: association with metastasis, recurrence, and prognosis. The Annals of Otology, Rhinology and Laryngology.

[94] Song L, Wang Z, Liu X (2013). MTA1: a prognosis indicator of postoperative patients with esophageal carcinoma. The Thoracic and Cardiovascular Surgeons.

[95] Iguchi H, Imura G, Toh Y, Ogata Y (2000). Expression of MTA1, a metastasis-associated gene with histone deacetylase activity in pancreatic cancer. International Journal of Oncology.

[96] Hofer MD, Chang MC, Hirko KA, Rubin MA, Nose V (2009). Immunohistochemical and clinicopathological correlation of the metastasis-associated gene 1 (MTA1) expression in benign and malignant pancreatic endocrine tumors. Modern Pathology.

[97] Hofer MD, Kuefer R, Varambally S, Li H, Ma J, Shapiro GI (2004). The role of metastasis-associated protein 1 in prostate cancer progression. Cancer Research.

[98] Dias SJ, Zhou X, Ivanovic M, Gailey MP, Dhar S, Zhang L (2013). Nuclear MTA1 overexpression is associated with aggressive prostate cancer, recurrence and metastasis in African Americans. Scientific Report.

[99] Bruning A, Makovitzky J, Gingelmaier A, Friese K, Mylonas I (2009). The metastasis-associated genes MTA1 and MTA3 are abundantly expressed in human placenta and chorionic carcinoma cells. Histochemistry and Cell Biology.

[100] Manavathi B, Peng S, Rayala SK, Talukder AH, Wang MH, Wang RA (2007). Repression of Six3 by a corepressor regulates rhodopsin expression. Proceedings of the National Academy of Sciences of the United States of America.

[101] Kumar R, Balasenthil S, Manavathi B, Rayala SK, Pakala SB (2010). Metastasis-associated protein 1 and its short form variant stimulates Wnt1 transcription through promoting its derepression from Six3 corepressor. Cancer Research.

[102] Pakala SB, Singh K, Reddy SD, Ohshiro K, Li DQ, Mishra L (2011). TGF-beta1 signaling targets metastasis-associated protein 1, a new effector in epithelial cells. Oncogene.

[103] Moon HE, Cheon H, Lee MS (2007). Metastasis-associated protein 1 inhibits p53-induced apoptosis. Oncology Reports.

[104] Choubey D (2000). P202: an interferon-inducible negative regulator of cell growth. Journal of Biological Regulators and Homeostatic Agents.

[105] Yan DH, Wen Y, Spohn B, Choubey D, Gutterman JU, Hung MC (1999). Reduced growth rate and transformation phenotype of the prostate cancer cells by an interferon-inducible protein, p202. Oncogene.

[106] Li DQ, Pakala SB, Reddy SD, Peng S, Balasenthil S, Deng CX (2013). Metastasis-associated protein 1 is an integral component of the circadian molecular machinery. Nature Communications.

[107] Pakala SB, Rayala SK, Wang RA, Ohshiro K, Mudvari P, Reddy SD (2013). MTA1 promotes STAT3 transcription and pulmonary metastasis in breast cancer. Cancer Research.

